# Influence of Mpv17 on Hair-Cell Mitochondrial Homeostasis, Synapse Integrity, and Vulnerability to Damage in the Zebrafish Lateral Line

**DOI:** 10.3389/fncel.2021.693375

**Published:** 2021-08-03

**Authors:** Melanie Holmgren, Lavinia Sheets

**Affiliations:** ^1^Department of Otolaryngology, Washington University School of Medicine, St. Louis, MO, United States; ^2^Department of Developmental Biology, Washington University School of Medicine, St. Louis, MO, United States

**Keywords:** Mpv17, mitochondrial homeostasis, hair cell, neuromast, damage

## Abstract

Noise exposure is particularly stressful to hair-cell mitochondria, which must produce enough energy to meet high metabolic demands as well as regulate local intracellular Ca^2+^ concentrations. Mitochondrial Inner Membrane Protein 17 (Mpv17) functions as a non-selective cation channel and plays a role in maintaining mitochondrial homeostasis. In zebrafish, hair cells in *mpv17^a9/a9^* mutants displayed elevated levels of reactive oxygen species (ROS), elevated mitochondrial calcium, hyperpolarized transmembrane potential, and greater vulnerability to neomycin, indicating impaired mitochondrial function. Using a strong water current to overstimulate hair cells in the zebrafish lateral line, we observed *mpv17^a9/a9^* mutant hair cells were more vulnerable to morphological disruption than wild type (WT) siblings simultaneously exposed to the same stimulus. To determine the role of mitochondrial homeostasis on hair-cell synapse integrity, we surveyed synapse number in *mpv17^a9/a9^* mutants and WT siblings as well as the sizes of presynaptic dense bodies (ribbons) and postsynaptic densities immediately following stimulus exposure. We observed mechanically injured *mpv17^a9/a9^* neuromasts were not more vulnerable to synapse loss; they lost a similar number of synapses per hair cell relative to WT. Additionally, we quantified the size of hair cell pre- and postsynaptic structures following stimulation and observed significantly enlarged WT postsynaptic densities, yet relatively little change in the size of *mpv17^a9/a9^* postsynaptic densities following stimulation. These results suggest chronically impaired hair-cell mitochondrial activity influences postsynaptic size under homeostatic conditions but does not exacerbate synapse loss following mechanical injury.

## Introduction

Hair cells, the sensory receptors of the inner ear, rely on mitochondria to generate energy to meet the high metabolic demands of mechanotransduction and synaptic transmission (Puschner and Schacht, 1997). Hair-cell mitochondria also produce reactive oxygen species (ROS) and contribute to the homeostatic control of intracellular Ca^2+^ (Matlib et al., 1998; Zenisek and Matthews, 2000; Collins et al., 2012; Wong et al., 2019). Disruption of mitochondrial dynamics can affect hair-cell vulnerability to damage from ototoxic drugs, as well as interfere with the maintenance of hair-cell synapses (Esterberg et al., 2014, 2016; Wong et al., 2019).

Zebrafish have emerged as a powerful tool to study the roles of mitochondria in hair-cell damage (Holmgren and Sheets, 2020). In addition to their ears, zebrafish have hair cells in their lateral line organs (neuromasts), which are composed of clusters of innervated hair cells and supporting cells. Zebrafish use their lateral line organs to detect local water currents and mediate behaviors such as escape responses and rheotaxis, or counter-flow swimming (Dijkgraaf, 1963; Olszewski et al., 2012; Suli et al., 2012; Stewart et al., 2013; Olive et al., 2016). Unlike cochlear hair cells, lateral-line hair cells are superficially located on the surface of the body and are therefore pharmacologically and optically accessible in an intact fish. Zebrafish became established as a model system for studying hair-cell development and function due to the identification of numerous conserved genes involved in hearing and balance (Nicolson, 2017).

Hearing loss is common in patients with mitochondrial disorders (Hsu et al., 2005). One gene that has been associated with mitochondrial disease in mammals and is conserved in zebrafish is *mpv17* (Müller et al., 1997; Krauss et al., 2013). *mpv17* encodes Mitochondrial Inner Membrane Protein 17 (Mpv17), which is a non-selective cation channel that modulates the mitochondrial potential and contributes to mitochondrial homeostasis (Antonenkov et al., 2015; Jacinto et al., 2021). Mice lacking Mpv17 show severe defects in the kidney, including glomerulosclerosis and nephrotic syndrome, and inner ear, including degeneration of outer hair cells and the stria vascularis (Müller et al., 1997; Meyer zum Gottesberge et al., 2001, 2012). In contrast, zebrafish lacking Mpv17 appear healthy and have normal life spans. Two zebrafish lines containing a spontaneous mutation in *mpv17* [*roy orbison* (*mpv17^a9/a9^*) and *transparent* (*mpv17^b18/b18^*)] both contain the same 19 bp deletion leading to aberrant splicing and a premature stop codon (Krauss et al., 2013; D’Agati et al., 2017). Notably, the *mpv17^a9/a9^* mutation is carried in the Casper strain of zebrafish, which are commonly used for imaging studies because they lack iridophores and thus have transparent skin (White et al., 2008; Martorano et al., 2019). Mpv17 has been shown in zebrafish to localize to mitochondria in multiple cell types, including lateral-line hair cells (Krauss et al., 2013). Although Casper fish are commonly used in research, how the loss of Mpv17 affects mitochondrial function in hair cells of the zebrafish lateral line has not yet been characterized. Moreover, as mitochondrial dysfunction is known to contribute to the pathologies underlying noise-induced hearing loss (Bottger and Schacht, 2013), we further wanted to examine the role of mitochondrial homeostasis in mechanically induced hair-cell damage.

In this study, we investigated how the loss of Mpv17 affects mitochondrial function in zebrafish lateral line hair cells as well as vulnerability to mechanical injury. In *mpv17^a9/a9^* hair cells, we observed elevated ROS and mitochondrial Ca^2+^, reduced FM1–43 uptake, and increased sensitivity to neomycin-induced hair-cell death. We have previously reported a protocol using a strong water current stimulus to induce mechanical damage to zebrafish lateral-line organs (Holmgren et al., 2021). When exposed to the same stimulus as WT siblings, mechanically overstimulated *mpv17^a9/a9^* neuromasts were more vulnerable to morphological disruption and hair-cell loss but showed similar degrees of de-innervation and synapse loss. Our results suggest that genetic disruption of mitochondrial homeostasis influences vulnerability to ototoxic or mechanically induced hair-cell death but does not exacerbate mechanically induced hair-cell synapse loss.

## Materials and Methods

### Zebrafish

All zebrafish experiments and procedures were performed in accordance with the Washington University Institutional Animal Use and Care Committee. Adult zebrafish were raised under standard conditions at 27–29°C in the Washington University Zebrafish Facility. Embryos were raised in incubators at 28°C in E3 media (5 mM NaCl, 0.17 mM KCl, 0.33 mM CaCl_2_, 0.33 mM MgCl_2_; Nüsslein-Volhard and Dahm, 2002) with a 14 h:10 h light:dark cycle. After 4 dpf, larvae were raised in 100–200 ml E3 media in 250-ml plastic beakers and fed rotifers daily. The sex of the animal was not considered for this study because sex cannot be determined in larval zebrafish.

The transgenic lines *TgBAC(neurod1:EGFP)*, *Tg(scm1:EGFP)*, and *Tg(myo6b:mitoGCaMP3)* were used in this study (Obholzer et al., 2008; Behra et al., 2009; Esterberg et al., 2014). Fluorescent transgenic larvae were identified at 3–5 days post-fertilization (dpf) under anesthesia with 0.01% tricaine in E3 media. The *TgBAC(neurod1:EGFP)*, *Tg(scm1:EGFP)*, and *Tg(myo6b:mitoGCaMP3)* lines were crossed into Casper compound mutants (*mitfa^w2/w2^*, *mpv17^a9/a9^*, White et al., 2008). Homozygous *mpv17^a9/a9^* mutants were identified at 3–5 dpf by phenotype under a brightfield dissecting microscope based on the severe reduction of iridophores in the eyes (D’Agati et al., 2017).

### Experimental Apparatus

This experimental device was previously described in Holmgren et al. (2021). In brief, 6-well plates containing larvae were clamped to a custom magnesium head expander (Vibration and Shock Technologies, Woburn, MA, USA) on a vertically-oriented Brüel + Kjær LDS Vibrator, V408 (Brüel and Kjær, Naerum, Denmark). The apparatus was housed in a custom sound-attenuation chamber. An Optiplex 3020 Mini Tower (Dell) with a NI PCIe-6321, X Series Multifunction DAQ (National Instruments) running a custom stimulus generation program (modified version of Cochlear Function Test Suite) was used to relay the stimulus signal to a Brüel + Kjær LDS PA100E Amplifier that drove a controlled 60 Hz vibratory stimulus along the plate’s dorsoventral axis (vertically). Two accelerometers (BU-21771, Knowles, Itasca, IL) were mounted to the head expander to monitor the vertical displacement of the plate. The output of the accelerometers was relayed through a custom accelerometer amplifier (EPL Engineering Core). A block diagram for the EPL Lateral Line Stimulator can be found here http://www.masseyeandear.org/research/otolaryngology/eaton-peabody-laboratories/engineering-core.

### Mechanical Overstimulation of Zebrafish Larvae

Larval zebrafish were exposed to strong water current as previously described (Holmgren et al., 2021). At 7 dpf, free-swimming zebrafish larvae were placed in untreated 6-well plates (Corning, Cat# 3736; well diameter: 34.8 mm; total well volume: 16.8 ml) with 6 ml E3 per well, pre-warmed to 28°C. Up to 20 larvae were placed in each well. Individual wells were sealed with Glad^®^ Press ‘n Seal plastic food wrap prior to placing the lid on the plate. An additional metal plate was fitted to the bottom of the multi-well dish to fill a small gap between the bottoms of the wells and the head expander.

Exposures (stimulus parameters: 60 Hz, 46.2 ± 0.3 m/s^2^) were conducted at room temperature (22–24°C) up to 2 h after dawn. Exposure consisted of 20 min of stimulation followed by a 10-minute break and 2 h of uninterrupted stimulation. During the entire duration of exposure, unexposed control fish were kept in the same conditions as noise-exposed fish i.e., placed in a multi-well dish and maintained in the same room as the exposure chamber. After exposure, larvae were immediately fixed for histology.

### Pharmacology

To assess hair-cell sensitivity to aminoglycosides, 5 dpf larvae were exposed to 1–100 μM neomycin (Sigma) in E3 for 30 min at 28°C. Larvae were then rinsed in E3 and allowed to recover for 2 h at 28°C before being fixed in 4% paraformaldehyde in PBS overnight at 4°C and mounted on slides with elvanol (13% w/v polyvinyl alcohol, 33% w/v glycerol, 1% w/v DABCO (1, 4 diazobicylo[2,2,2] octane) in 0.2 M Tris, pH 8.5).

To verify that entry of MitoTracker dyes into hair cells was not dependent on mechanotransduction, larvae were treated with BAPTA, which breaks hair-cell tip links. Seven dpf larvae were exposed to 5 mM BAPTA (Invitrogen) in E3 for 10 min, then rinsed in E3. MitoTracker probes were calibrated by treating fish with cyclosporin A (TCI America). Larvae were exposed to 200 nM cyclosporin A alone in E3 with 0.1% DMSO for 5 min prior to co-exposure with MitoTracker probes and drug for 30 min.

### Live Hair-Cell Labeling

Hair cell nuclei were specifically labeled by briefly exposing free-swimming larvae to 4′,6-Diamidino-2-Phenylindole (DAPI; Invitrogen/ThermoFisher) diluted to 2.5 μg/ml in E3. Prior to exposure to mechanical stimulation or incubation with other indicators, larvae were exposed to DAPI working solution for 4 min, then rinsed three times in E3.

CellROX Green (Invitrogen) was used to quantify levels of ROS in hair cells. Larvae were exposed to 5 μM CellROX Green in E3 for 30 min at 28°C, protected from light. Larvae were then rinsed twice in E3 and prepared for live imaging. Larvae were maintained in the dark prior to imaging.

The fixable vital dye FM1-43FX (Invitrogen) was used to quantify hair-cell mechanotransduction as previously described (Holmgren et al., 2021). In brief, 7 dpf larvae were exposed to 3 μM FM1-43FX in E3 for 20 s then rinsed in E3. Larvae were then fixed (4% paraformaldehyde, 4% sucrose, 150 μM CaCl_2_ in 0.1 M phosphate buffer) overnight at 4°C and mounted on slides with elvanol.

We chose to use MitoTracker Red CMXRos and MitoTracker Deep Red (Invitrogen) to measure mitochondrial membrane potential because they are well retained after fixation, which allowed a higher temporal resolution than probes that require live imaging (Pendergrass et al., 2004; Mot et al., 2016). 7 dpf larvae were exposed to 500 nM MitoTracker Red CMXRos and 500 nM MitoTracker Deep Red concurrently in E3 for 30 min at 28°C, protected from light. Larvae were then rinsed in E3, fixed in 4% paraformaldehyde in PBS overnight at 4°C, and mounted on slides with elvanol.

### Whole-Mount Immunohistochemistry

Larvae were briefly sedated on ice and fixed (4% paraformaldehyde, 4% sucrose, 150 μM CaCl_2_ in 0.1 M phosphate buffer) for 5 h at 4°C. Larvae were then permeabilized in ice-cold acetone, blocked (2% goat serum, 1% bovine serum albumin, 1% DMSO in PBS), and incubated with primary antibodies diluted in blocking buffer. The following commercial primary antibodies were used in this study: GFP (1:500; Aves Labs, Inc; Cat# GFP-1020), Parvalbumin (1:2,000; Thermo Fisher; Cat# PA1-933), Parvalbumin (1:2,000; Abcam; Cat# ab11427), Parvalbumin (1:500; Sigma-Aldrich; Cat# P3088), MAGUK (K28/86; 1:500; NeuroMab, UC Davis; Cat# 75-029), CtBP (1:2,000; Santa Cruz Biotechnology Cat# sc-55502. Custom affinity-purified antibody generated against *Danio rerio* Ribeye b was also used (mouse IgG2a; 1:2,000; Sheets et al., 2011). Following primary antibody incubation, larvae were washed and incubated with diluted secondary antibodies conjugated to Alexa Fluor 488, Alexa Fluor 546, Dylight 549, Alexa Fluor 555, and Alexa Fluor 647 (Invitrogen). Larvae were then counterstained with DAPI (Sigma) and mounted on slides with elvanol.

### Live Imaging

Live imaging of zebrafish larvae was performed as previously described (Holmgren et al., 2021). In brief, zebrafish larvae were anesthetized with 0.01% tricaine in E3, then mounted lateral-side up on a thin layer of 1.5–2% low-melt agarose in a tissue culture dish with a cover-glass bottom (World Precision Instruments) and covered in E3 media. Z-stack images with a z step of 1 μm (CellROX) or 0.5 μm (mito-GCaMP3) were acquired *via* an ORCA-Flash 4.0 V3 camera (Hamamatsu) using a Leica DM6 Fixed Stage microscope with an X-Light V2TP spinning disc confocal (60 micron pinholes) and a 63×/0.9 N.A. water immersion objective. Z- acquisition parameters w/ X-light spinning disc: 488 laser “20% power”, 150 ms per frame. The perimeter of each neuromast for subsequent analysis was captured using differential interference contrast imaging following fluorescent image acquisition. Image acquisition was controlled by MetaMorph software.

### Confocal Imaging of Fixed Specimens

Fixed sample images were acquired using an LSM 700 laser scanning confocal microscope with a 63× 1.4 NA Plan-Apochromat oil-immersion objective (Carl Zeiss). Confocal stacks were collected with a z step of 0.3 μm over 7–10 μm with pixel size of 100 nm (x–y image size 51 × 51 μm). Acquisition parameters were established using the brightest control specimen such that just a few pixels reached saturation in order to achieve the greatest dynamic range in our experiments. For quantitative measurements such as particle volume or fluorescence intensity, parameters including gain, laser power, scan speed, dwell time, resolution, and zoom, were kept consistent between comparisons.

### Confocal Image Processing and Analysis

Analysis was performed on blinded images of posterior lateral-line neuromasts L3, L4, and L5. Digital images were processed using ImageJ software (Schneider et al., 2012). To correct for drift in the z direction, images were adjusted using StackReg when appropriate (Thevenaz et al., 1998). Subsequent image processing for display within figures was performed using Illustrator software (Adobe).

To measure the volume of synaptic puncta, raw images containing a single immunolabel were subtracted for background using a 20-pixel rolling ball radius. Whole neuromasts were delineated based on the Parvalbumin label in maximum-intensity projections using the freehand selection and “synchronize windows” tools. Puncta were defined as regions of immunolabel with pixel intensity above a determined threshold: threshold for Ribeye label was calculated using the Isodata algorithm (Ridler and Calvard, 1978) on maximum-intensity projections, the threshold for MAGUK label was calculated as the product of seven times the average pixel intensity of the whole NM region in a maximum-intensity projection. A common threshold calculation for both channels could not be used due to differences in background staining intensity between Ribeye and MAGUK labels. Particle volume was measured using the 3D object counter using a lower threshold and minimum particle size of 10 voxels (Bolte and Cordelieres, 2006). To normalize for differences in intensity across experimental trials, volume measurements were normalized to the median WT control volume for each trial for each individual channel. Intact synapses were manually counted and defined as colocalized or adjoining MAGUK and Ribeye or CtBP puncta. The number of intact synapses per hair cell was approximated by dividing the number of synapses by the number of hair cells in the neuromast.

To measure the fluorescence intensity of indicators across whole neuromasts, images containing single channels were background-subtracted using a rolling ball radius of the following sizes: images containing celllROX label or mitoGCaMP3 (50-pixel), images containing FM1-43FX label (100-pixel), and images containing individual MitoTracker labels (200-pixel). Whole neuromasts were delineated based on DIC images (celllROX label and mitoGCaMP3) or hair cell specific DAPI label in maximum-intensity projections, and the mean intensity of the indicator was measured. Measurements from each experimental trial were normalized to the WT control median value.

### Statistical Analysis

Statistical analyses were performed using Prism 9 (Graphpad Software Inc). Datasets were confirmed for normality using the D’Agostino-Pearson test when appropriate. Statistical significance between two groups was determined by an unpaired Student’s *t*-test or a Mann–Whitney *U* test, depending on the normality of the dataset. Statistical significance between multiple groups with normal distributions was determined by one-way ANOVA and appropriate *post hoc* tests, and statistical significance between multiple groups with non-normal distributions was determined by a Kruskal-Wallis test and appropriate *post hoc* tests. For datasets dependent on multiple independent variables, statistical significance was determined using two-way ANOVA and appropriate *post hoc* tests.

Individual afferent neurons innervate multiple hair cells per neuromast as well as multiple neuromasts per fish. As such, events such as de-innervation and synapse loss could not be considered independent. To account for this influence, measurements from multiple neuromasts (L3–L5), including hair-number, synapse number, and hair-cell innervation, were recorded from individual fish and the average values for each fish were used for our analysis.

## Results

### Mitochondrial Homeostasis Is Disrupted in *mpv17^a9/a9^* Hair Cells

**Figure 1:**
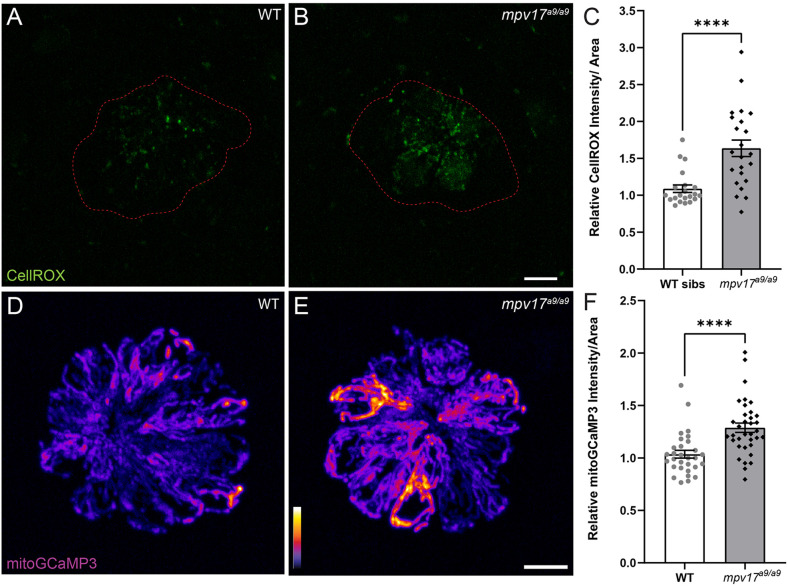
Mitochondrial homeostasis in WT and *mpv17^a9/a9^* neuromasts. **(A,B)** Maximum-intensity projections of confocal images showing CellROX Green staining in WT siblings **(A)** and *mpv17^a9/a9^* **(B)** neuromasts. Neuromast boundaries were delineated based on DIC images (not shown). **(C)** Mean CellROX intensity is elevated in *mpv17^a9/a9^* neuromasts (****P < 0.0001). *n* = 21–23 neuromasts; *N* = 2 experimental trials. **(D,E)** Maximum-intensity projections of confocal images showing mitoGCaMP3 fluorescence in WT **(D)** and *mpv17^a9/a9^* **(E)** neuromasts. **(F)** Mean mitoGCaMP3 intensity is increased in *mpv17^a9/a9^* neuromasts (****P < 0.0001). *n* = 30–35 neuromasts (L3, L4, and L5); *N* = 2 experimental trials. Scale bars: 5 μm. Error bars: SEM.

It has been previously reported that zebrafish lacking Mpv17 have impaired mitochondrial function and that Mpv17 protein localizes to hair-cell mitochondria (Krauss et al., 2013; Martorano et al., 2019). We thus characterized how the loss of Mpv17 affected mitochondrial homeostasis in hair cells of the lateral line. To quantify ROS levels in lateral-line hair cells, we exposed *mpv17^a9/a9^* larvae and their WT siblings to the probe CellROX Green (Figures 1A,B). We observed increased fluorescence in *mpv17^a9/a9^* neuromasts relative to WT, indicating elevated ROS in *mpv17^a9/a9^* hair cells (Figure 1C; Mann-Whitney test ****P < 0.0001). We then quantified baseline mitochondrial Ca^2+^ levels using the genetically encoded indicator mitoGCaMP3 (Figures 1D,E; Esterberg et al., 2014). Relative to WT siblings, *mpv17^a9/a9^* neuromasts had increased mitoGCaMP3 fluorescence, indicative of elevated mitochondrial Ca^2+^ (Figure 1F; Mann-Whitney test ****P < 0.0001).

**Figure 2:**
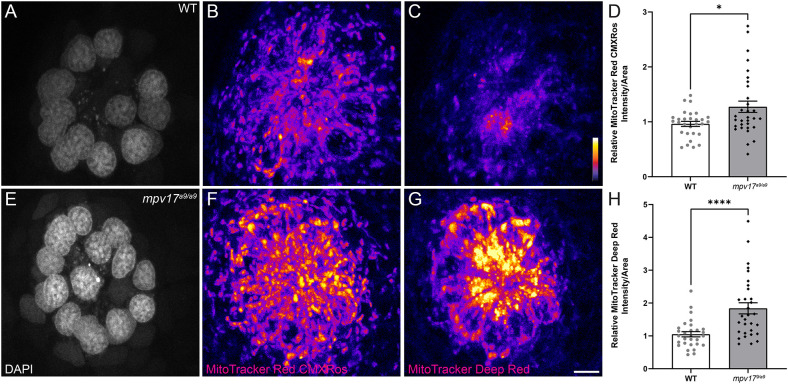
*mpv17^a9/a9^* hair cells have hyperpolarized mitochondria. **(A–C, E–G)** Maximum-intensity projections of confocal images displaying WT **(A–C)** and *mpv17^a9/a9^* **(E–G)** neuromasts with DAPI-labeled hair cells **(A–E)** and staining with MitoTracker Red CMXRos **(B–F)** and MitoTracker Deep Red **(C–G)**. **(D–H)** Mean intensities for both MitoTracker Red CMXRos **(D)** and MitoTracker Deep Red **(H)** are elevated in *mpv17^a9/a9^* neuromasts (*P = 0.0469; ****P < 0.0001). *n* = 29–31 neuromasts (L3, L4, and L5); *N* = 4 experimental trials. Scale bar: 5 μm. Error bars: SEM.

It has been shown in murine fibroblasts that loss of Mpv17 results in hyperpolarized mitochondria (Antonenkov et al., 2015). To measure mitochondrial membrane potential (ΔΨ_m_) of lateral-line hair cells, we exposed *mpv17^a9/a9^* and sibling larvae to the dyes MitoTracker Red CMXRos and MitoTracker Deep Red (Figures 2A–C, E–G). Both of these dyes are well-retained after fixation and their accumulation in mitochondria is dependent on ΔΨ_m_ (Pendergrass et al., 2004; Mot et al., 2016). We verified that these probes could be used to detect hyperpolarized ΔΨ_m_ by treating WT larvae with cyclosporin A (Supplementary Figures 1A,B). While MitoTracker uptake is not specific to hair cells, we also verified that MitoTracker entry into hair cells is not dependent on hair-cell mechanotransduction by briefly treating larvae with BAPTA to disrupt tip links prior to MitoTracker exposure (Supplementary Figures 1A,B). With both probes, we observed increased fluorescence in *mpv17^a9/a9^* neuromasts relative to WT, indicating *mpv17^a9/a9^* hair cells have hyperpolarized mitochondria (Figures 2D,H; *P = 0.0469 MitoTracker Red CMXRos; ****P < 0.0001 MitoTracker Deep Red).

**Figure 3:**
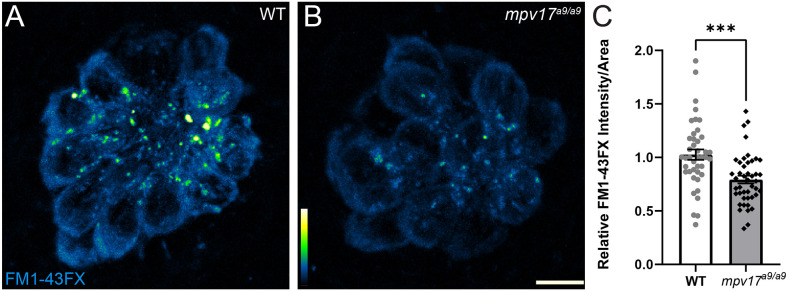
FM1-43 uptake is reduced in *mpv17^a9/a9^* hair cells. **(A,B)** Maximum-intensity projections of confocal images showing WT **(A)** and *mpv17^a9/a9^* **(B)** neuromasts exposed to FM1-43FX. **(C)** Mean intensity of FM1-43FX is reduced in *mpv17^a9/a9^* neuromasts (***P = 0.0002). *n* = 42–44 neuromasts (L3, L4, and L5); *N* = 4 experimental trials. Scale bar: 5 μm. Error bars: SEM.

To determine the effect of Mpv17 deficiency on hair-cell function, we next exposed *mpv17^a9/a9^* and WT larvae to the vital dye FM1–43FX (Figures 3A,B). Labeling of hair cells following brief exposure to FM1–43 occurs *via* active mechanotransduction, and reduced labeling indicates reduced driving force for cations through mechanotransduction channels (Gale et al., 2001; Toro et al., 2015). We observed significantly reduced uptake of this dye in *mpv17^a9/a9^* neuromasts relative to WT (Figure 3C; Unpaired *t*-test ***P = 0.0002), suggesting impaired mechanotransduction in *mpv17^a9/a9^* hair cells. Collectively, these results support that mitochondrial homeostasis is disrupted in *mpv17^a9/a9^* hair cells, and that hair-cell transduction is also reduced.

### *mpv17^a9/a9^* Hair Cells Are More Susceptible to Neomycin-Induced Death

**Figure 4:**
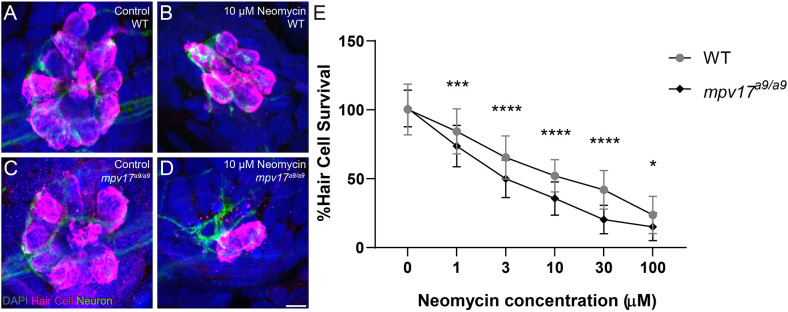
*mpv17^a9/a9^* larvae are more sensitive to neomycin-induced hair-cell loss. **(A–D)** Maximum-intensity projections of confocal images of WT **(A,B)** and *mpv17^a9/a9^* **(C,D)** control neuromasts **(A–C)** or exposed to 10 μM neomycin **(B,D)**. Hair cells were visualized with immunolabel of Parvalbumin (magenta), afferent neurites are expressing GFP (green), and all cell nuclei are labeled with DAPI (blue). **(E)** Dose-response curves showing hair cell survival as a percentage of control in both WT and *mpv17^a9/a9^* neuromasts (*P = 0.0115; ***P = 0.0001; ****P < 0.0001). *n* = 67–86 neuromasts (L3, L4, and L5); *N* = 3 experimental trials. Scale bar: 5 μm. Error bars: SD.

A recent study demonstrated that zebrafish mutants with elevated ROS are more vulnerable to neomycin-induced hair-cell loss (Alassaf et al., 2019). It has also been shown that elevated mitochondrial Ca^2+^ or ΔΨ_m_ increases sensitivity to neomycin, and that a hair cell’s cumulative mitochondrial activity can predict its sensitivity to neomycin (Esterberg et al., 2014; Pickett et al., 2018). On the other hand, it is known that neomycin enters hair cells through mechanotransduction channels, and blocking mechanotransduction can reduce neomycin sensitivity (Owens et al., 2009; Alharazneh et al., 2011; Hailey et al., 2017). We therefore exposed *mpv17^a9/a9^* and WT sibling larvae to a range of doses of neomycin to determine whether *mpv17^a9/a9^* larvae are more or less sensitive to neomycin-induced hair-cell death. At all doses tested, *mpv17^a9/a9^* neuromasts lost significantly more hair cells following exposure than WT (Figure 4; repeated measure two-way ANOVA ***P = 0.001 1 μM; ****P < 0.0001 3 μM; ****P <0.0001 10 μM; ****P < 0.0001 30 μM; *P = 0.0115 100 μM). Thus, loss of Mpv17 increases sensitivity to neomycin-induced hair-cell death.

### *mpv17^a9/a9^* Hair Cells Are More Vulnerable to Mechanically-Induced Morphological Disruption

**Figure 5:**
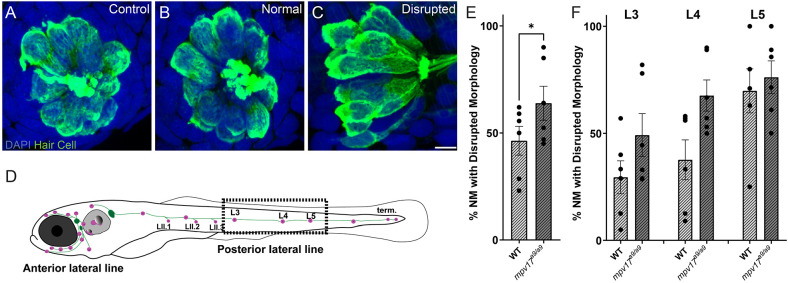
Mechanical overstimulation results in morphological disruption of neuromasts more frequently in *mpv17^a9/a9^* larvae than in WT. **(A–C)** Maximum-intensity projections of confocal images showing hair cells labeled with Parvalbumin and all nuclei labeled with DAPI in control neuromasts **(A)** and strong water current exposed neuromasts with normal **(B)** or disrupted **(C)** morphology. **(D)** Schematic of larval zebrafish indicating the placement of neuromasts (pink dots) and afferent nerves (green lines). Neuromasts L3, L4, and L5 (dashed rectangle) were examined in this study. **(E,F)** Quantification of disrupted neuromasts, both overall **(E)** and separated by position on the body **(F)**. Each point indicates the percentage of neuromasts with disrupted morphology in a single experimental trial. The frequency of disrupted neuromasts was higher in the more posterior L5 neuromasts; *mpv17^a9/a9^* neuromasts showed disrupted morphology more frequently than WT (*P = 0.0232). *n* = 9–64 neuromasts (L3, L4, and L5) per trial; *N* = 6 experimental trials. Scale bar: 5 μm. Error bars: SEM.

We have previously reported a protocol to mechanically overstimulate zebrafish lateral line organs using a strong water current (Holmgren et al., 2021). This stimulation resulted in phenotypes including mechanical disruption of neuromast morphology, loss of hair cells, neurite retraction, and loss of hair-cell synapses. To determine the effects of impaired mitochondrial homeostasis on mechanically induced lateral-line damage, we exposed 7-day-old *mpv17^a9/a9^* larvae and WT siblings to strong water current then fixed them for immunohistochemical labeling of hair cells, neurites, and synaptic components (Figures 5A–C). As indicated in our previous study, image analysis was performed on blinded samples. As in our previous study, in fish exposed to strong water current, we observed two distinct morphological profiles of the neuromasts: “normal” in which the neuromasts appeared identical to controls with the hair cells arranged in a typical rosette structure (Figure 5B), or “disrupted,” in which the neuromasts were displaced on their sides with elongated and misshapen hair cells and the apical ends of the hair cells oriented posteriorly (Figure 5C). In stimulus exposed WT fish, we observed disrupted morphology in 46% of the neuromasts surveyed (Figure 5E). We observed this morphological change more frequently in the more posterior L5 neuromasts compared to the more anterior L3 neuromasts (Figure 5F; 29% L3; 37% L4; 70% L5). We observed in *mpv17^a9/a9^* larvae a similar trend of increased morphological disruption in the more posterior neuromasts (49% L3; 68% L4; 76% L5), however, the frequency of disrupted neuromasts was higher in mechanically injured *mpv17^a9/a9^* larvae compared to WT siblings concurrently exposed to the same stimulus (Figure 5E; 64%; Paired *t*-test *P = 0.0232). Thus, *mpv17^a9/a9^* neuromasts appear to be more vulnerable to morphological disruption resulting from mechanical injury.

**Figure 6:**
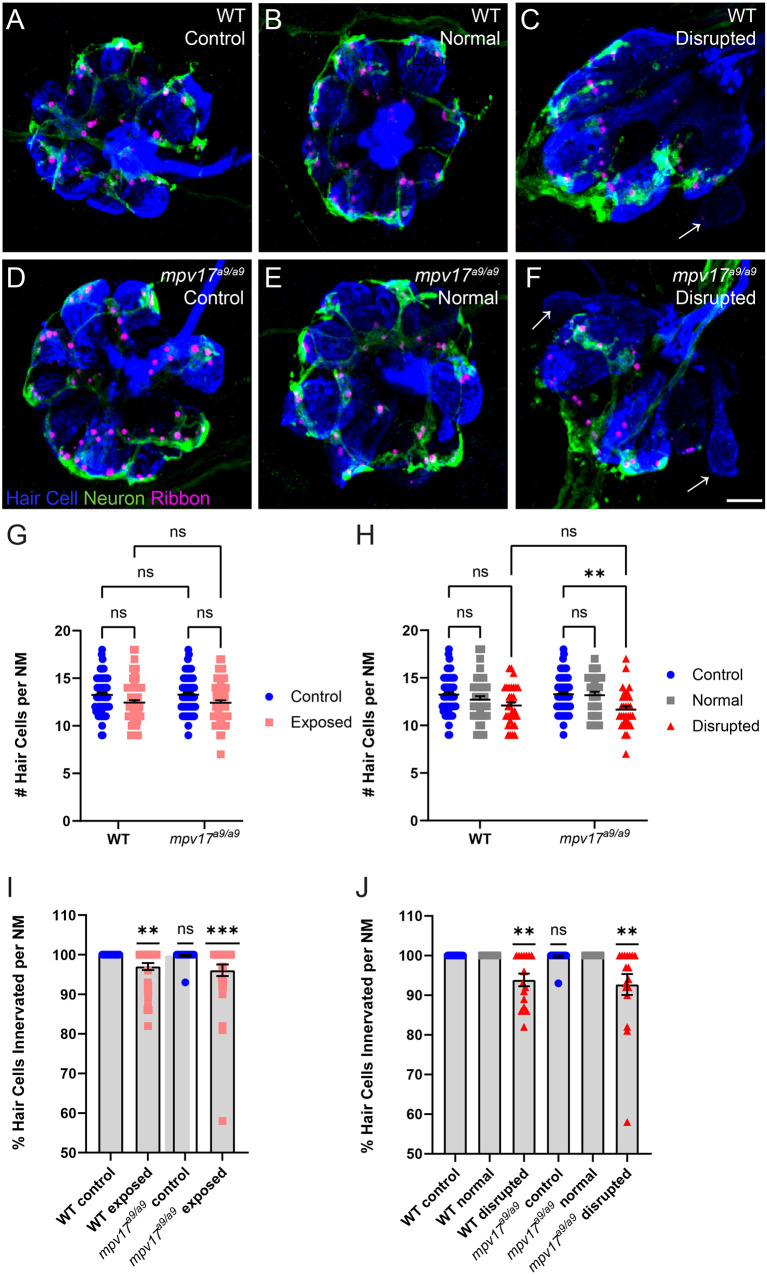
WT and *mpv17^a9/a9^* neuromasts show loss of hair cells and afferent innervation following mechanical overstimulation. **(A–F)** Maximum-intensity projections of confocal images showing neuromasts with Parvalbumin-labeled hair cells (blue) and Ribeye b-labeled presynaptic ribbons (magenta). Neurod:GFP-labeled afferent neurites are also shown (green). Unexposed control neuromasts are shown in (**A**; WT) and (**D**; *mpv17^a9/a9^*); exposed neuromasts with normal morphology are shown in (**B**; WT) and (**E**; *mpv17^a9/a9^*); and exposed neuromasts with disrupted morphology are shown in (**C**; WT) and (**F**; *mpv17^a9/a9^*). Arrows indicate hair cells lacking afferent innervation. **(G,H)** Quantification of average hair cells per neuromast shows a trend of hair cell loss in exposed neuromasts **(G)**, which is specific to disrupted neuromasts (**H**; **P = 0.0037). *n* = 32–66 fish (neuromasts L3, L4, and L5); *N* = 9 experimental trials. **(I,J)** The average percentage of hair cells with GFP-labeled contacts. We observed significant neurite retraction [**I**; **P = 0.0020 (WT); ***P = 0.0010 (*mpv17^a9/a9^*)], which is also specific to disrupted neuromasts only [**J**; **P = 0.0039 (WT); **P = 0.0020 (*mpv17^a9/a9^*)]. *n* = 15–30 fish (neuromasts L3, L4, and L5); *N* = 6 experimental trials. Scale bar: 5 μm. Error bars: SEM. “ns” = not significant.

We then compared hair-cell numbers between mechanically injured *mpv17^a9/a9^* and WT neuromasts. Both WT and *mpv17^a9/a9^* larvae sibling fish exhibited a slight decrease in the number of hair cells per neuromast (Figure 6G; Repeated measure two-way ANOVA *P* = 0.1033 WT; *P* = 0.0764 *mpv17^a9/a9^*). In both groups, this reduction in hair-cell number was specific to disrupted neuromasts; hair-cell numbers in exposed neuromasts with “normal” hair-cell morphology were comparable to control (Figure 6H; repeated measure two-way ANOVA *P* = 0.7933 WT normal; *P* = 0.0969 WT disrupted; *P* > 0.9999 *mpv17^a9/a9^* normal; **P < 0.0037 *mpv17^a9/a9^* disrupted). We also observed a significant reduction in the percentage of hair cells per neuromast with *neurod:EGFP* labeled afferent contacts in both WT and *mpv17^a9/a9^* neuromasts (Figure 6I; one sample Wilcoxon test **P = 0.0020 WT; ***P = 0.0010 *mpv17^a9/a9^*). Similar to the reduction in hair-cell number, this neurite retraction phenotype was evident only in “disrupted” neuromasts of both groups (Figure 6J; one sample Wilcoxon test **P = 0.0039 WT disrupted; **P = 0.0020 *mpv17^a9/a9^* disrupted). Taken together, these results demonstrate that *mpv17^a9/a9^* neuromasts are more susceptible to mechanically induced morphological disruption, but that impaired mitochondrial function does not affect sensitivity to afferent retraction resulting from mechanical injury.

### WT and *Mpv17^a9/a9^* Hair Cells Show Comparable Synapse Loss Following Mechanical Overstimulation

**Figure 7:**
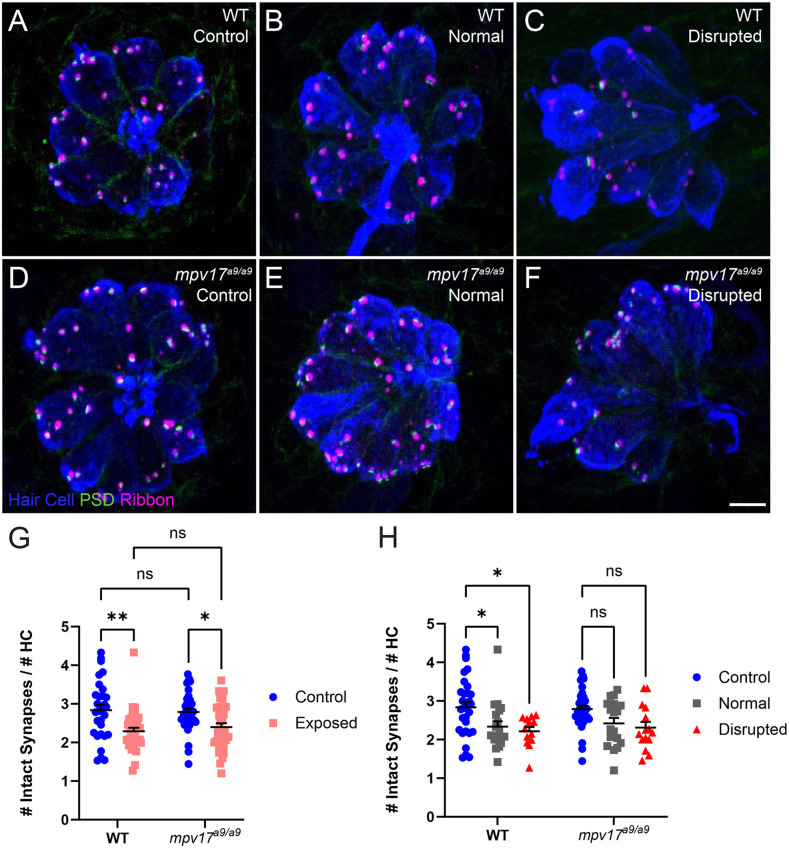
Both WT and *mpv17^a9/a9^* neuromasts experience mechanically induced hair-cell synapse loss. **(A–C)** Maximum-intensity projections of confocal images showing neuromasts with Parvalbumin-labeled hair cells (blue), Ribeye b-labeled presynaptic ribbons (magenta), and MAGUK-labeled PSD (green). Unexposed control neuromasts are shown in (**A**; WT) and (**D**; *mpv17^a9/a9^*); exposed neuromasts with normal morphology are shown in (**B**; WT) and (**E**; *mpv17^a9/a9^*); and exposed neuromasts with disrupted morphology are shown in (**C**; WT) and (**F**; *mpv17^a9/a9^*). **(G,H)** Quantification of average numbers of intact synapses per hair cell. Each data point refers to the average number of intact synapses per hair cell in one neuromast per fish. Synapse number is reduced in both WT and *mpv17^a9/a9^* neuromasts (**G**; **P = 0.0020 WT; *P = 0.0313 *mpv17^a9/a9^*). This reduction was consistent in both normal and disrupted neuromasts (**H**; *P = 0.0483 WT normal; *P = 0.0272 WT disrupted). *n* = 13–36 fish (neuromasts L3, L4, and L5); *N* = 7 experimental trials. Scale bar: 5 μm. Error bars: SEM. “ns” = not significant. PSD, postsynaptic density.

Recent studies indicate hair-cell mitochondrial activity plays a key role in synaptic maintenance (Wang et al., 2018; Wong et al., 2019). We have previously shown that mechanical overstimulation resulted in the loss of hair-cell synapses, as well as changes in synapse morphology (Holmgren et al., 2021). To determine the effect of impaired mitochondrial function on synapse number following mechanical overstimulation, we exposed *mpv17^a9/a9^* fish and their WT siblings, then immunolabeled synaptic components and quantified intact synapses, defined as presynaptic ribbons colocalized with postsynaptic densities (PSD; Figures 7A–F). In mechanically overstimulated WT neuromasts, we observed a statistically significant decrease in the number of intact synapses per hair cell (Figure 7G; repeated measure two-way ANOVA **P = 0.0020 WT). In agreement with our previous study, this loss of synapses occurred in all exposed neuromasts i.e., both normal and disrupted morphologies (Figure 7H; repeated measure two-way ANOVA *P = 0.0483 WT normal; *P = 0.0272 WT disrupted), suggesting that synapse loss may be a consequence of hair-cell overstimulation rather than physical injury. In mechanically overstimulated *mpv17^a9/a9^* fish, we observed a similar reduction in synapse number (Repeated measure two-way ANOVA *P = 0.0313), and this trend of synapse loss occurred in all exposed neuromasts (Repeated measure two-way ANOVA *P* = 0.2806 *mpv17^a9/a9^* normal; *P* = 0.1048 *mpv17^a9/a9^* disrupted). The similarity in synapse loss between mutants and WT siblings suggests loss of Mpv17 does not dramatically affect mechanically induced synapse loss. Also notable was that unexposed control WT and *mpv17^a9/a9^* fish had a comparable number of synapses per hair cell (Figure 7G; *P* = 0.9893) indicating that chronic mitochondrial dysfunction in *mpv17^a9/a9^* hair cells does not affect synaptic maintenance.

**Figure 8:**
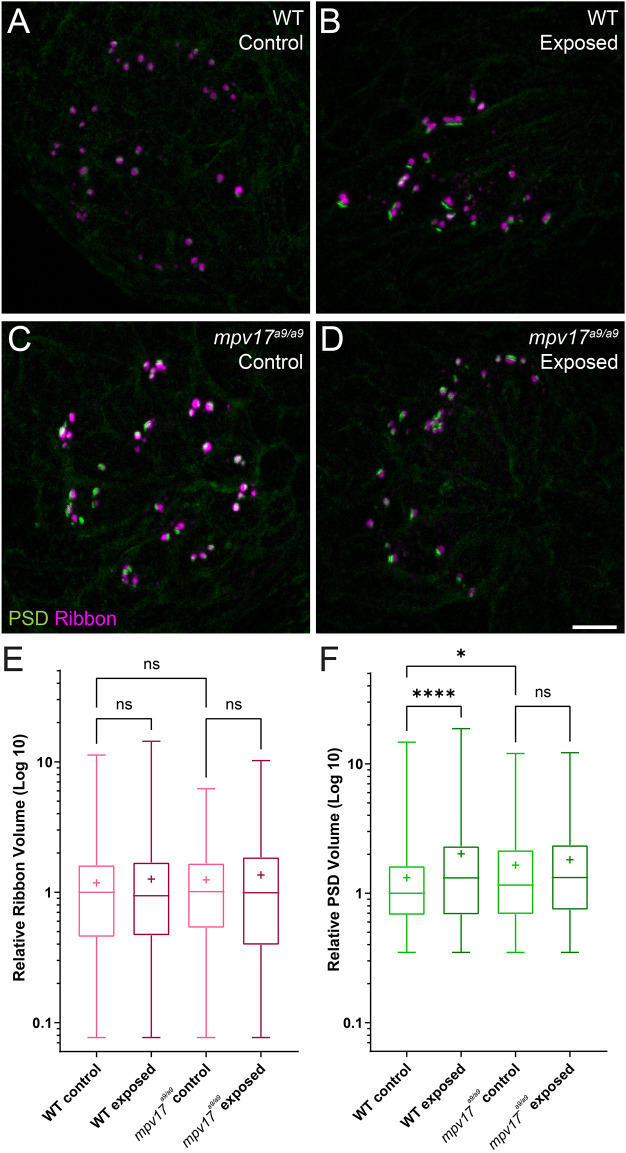
PSDs are enlarged in *mpv17^a9/a9^* neuromasts but do not significantly change upon mechanical overstimulation. **(A–D)** Maximum-intensity projections of confocal images of WT **(A,B)** and *mpv17^a9/a9^* **(C,D)** neuromasts, either unexposed **(A–C)** or exposed to strong water current **(B,D)**. **(E)** Quantification of presynaptic ribbon volume relative to unexposed WT control. *n* = 1013–1087 presynaptic particles; *N* = 7 experimental trials. **(F)** Quantification of PSD volume relative to WT control. PSDs are enlarged in unexposed *mpv17^a9/a9^* neuromasts and mechanically overstimulated WT neuromasts, but there is no significant change in PSD size in mechanically overstimulated *mpv17^a9/a9^* neuromasts (****P < 0.0001 WT exposed; *P = 0.0100 *mpv17^a9/a9^* control). *n* = 486–683 PSD particles from neuromasts L3, L4, and L5; *N* = 5 experimental trials. Scale bar: 5 μm. Whiskers: min. to max. values; “+” indicates mean value. “ns” = not significant.

Studies in mammals have shown that noise exposures modulate the sizes of inner hair cell pre- and postsynaptic components (Song et al., 2016; Kim et al., 2019). We have also previously demonstrated that mechanical overstimulation of the lateral line resulted in enlarged PSDs (Holmgren et al., 2021). We thus measured the relative volumes of presynaptic ribbons and PSDs in *mpv17^a9/a9^* larvae and WT siblings following mechanical overstimulation. We observed no significant change in the sizes of presynaptic ribbons in either WT or *mpv17^a9/a9^* exposed neuromasts immediately following exposure (Figure 8E; Kruskal-Wallis test *P* = 0.6165). When we compared PSD volumes, we observed enlarged PSD in unexposed *mpv17^a9/a9^* control neuromasts relative to WT (Figures 8C–F; Dunn’s multiple comparison test *P = 0.0100). In mechanically overstimulated WT neuromasts, there was a dramatic increase in PSD size relative to control (Figures 8B–F; Dunn’s multiple comparison test ****P < 0.0001). By contrast, in exposed *mpv17^a9/a9^* neuromasts, there was a modest, nonsignificant increase in PSD size relative to control, and the increase was less dramatic than in WT (Figures 8D–F; Dunn’s multiple comparison test *P* = 0.1787). These results reveal *mpv17^a9/a9^* mutant hair cells possess somewhat enlarged PSDs under homeostatic conditions and undergo less dramatic changes in PSD size following mechanical overstimulation relative to WT.

## Discussion

### Effects of Mpv17 Deficiency in Zebrafish Lateral-Line Hair Cells

In this study, we have characterized the effects of Mpv17 deficiency on lateral-line hair cells, both under homeostatic conditions and in response to mechanical overstimulation. Mitochondrial homeostasis appeared to be disrupted, as we observed in *mpv17^a9/a9^* mutant hair cells elevated ROS and mitochondrial Ca^2+^, as well as hyperpolarized ΔΨ_m_. Mpv17-deficient hair cells showed reduced FM1–43 uptake and were more susceptible to neomycin-induced death. Following mechanical overstimulation, *mpv17^a9/a9^* neuromasts were more vulnerable to morphological disruption than WT siblings, but showed similar degrees of de-innervation and synapse loss.

To our knowledge, while the transparent Casper mutant line of zebrafish is commonly used in research, this is the first investigation of the role of Mpv17 in lateral-line hair-cell mitochondrial dynamics. Mpv17 function has been studied more extensively in mammalian models (Müller et al., 1997; Binder et al., 1999; Meyer zum Gottesberge et al., 2001, 2012). In mice, loss of Mpv17 results in severe defects in the kidney and inner ear. In humans, severe *MPV17* deficiency has been associated with a hepatocerebral form of mitochondrial DNA depletion syndrome which results in death due to liver failure at young ages, while less severe mutations have been linked to juvenile-onset peripheral neuropathy (Spinazzola et al., 2008; El-Hattab et al., 2018; Baumann et al., 2019). The phenotypes observed in *mpv17^a9/a9^* zebrafish are much milder than those reported in mammals, as homozygous mutants appear healthy and have normal lifespans. Paralogous genes in zebrafish may provide compensatory mechanisms for the loss of Mpv17 (Glasauer and Neuhauss, 2014). Zebrafish have two *mpv17*-like genes, *mpv17l* and* mpv17l2*, and *mpv17l2* is upregulated in *mpv17^a9/a9^* fish (Krauss et al., 2013; Martorano et al., 2019). While it is known that *mpv17l2* is strongly expressed in the larval zebrafish liver (Thisse and Thisse, 2004), it remains to be determined whether *mpv17l2* is expressed specifically in hair cells.

### Mitochondrial Homeostasis in *Mpv17^a9/a9^* Hair Cells

Mpv17-deficient hair cells showed elevated ROS and mitochondrial Ca^2+^, as well as a more negative ΔΨ_m_ (Figures 1, 2). Mitochondrial Ca^2+^, ROS production, and ΔΨ_m_ are tightly linked in the cell (Brookes et al., 2004; Adam-Vizi and Starkov, 2010; Ivannikov and Macleod, 2013; Esterberg et al., 2014; Gorlach et al., 2015). ROS are generated in the mitochondria as a consequence of oxidative phosphorylation, which depends on a negative ΔΨ_m_. Negative ΔΨ_m_ is also maintained by removing protons from the mitochondrial matrix as electrons flow through the electron transport chain. A more negative ΔΨ_m_ thus results in more ROS (Kann and Kovacs, 2007; Zorov et al., 2014). Mitochondrial Ca^2+^ regulates oxidative phosphorylation, so the elevated mitochondrial Ca^2+^ observed in *mpv17^a9/a9^* hair cells also contributes to increased ROS and ΔΨ_m_ (Brookes et al., 2004).

Similar hair-cell phenotypes were recently reported in *pappaa^p170^* mutant zebrafish: *pappaa^p170^* hair cells had elevated mitochondrial Ca^2+^, elevated ROS, and hyperpolarized mitochondria (Alassaf et al., 2019). Additionally, these mutants were more sensitive to neomycin-induced hair-cell loss, similar to what we observed in *mpv17^a9/a9^* neuromasts (Figure 4). One notable difference between *pappaa^p17^*^0^ a*nd mpv17^a9/a9^* mutant hair cells is that the significantly reduced FM1–43 uptake we observed in *mpv17^a9/a9^* mutants (Figure 3) was not observed in *pappaa^p170^* mutants, indicating that the driving force for cations through mechanotransduction channels is decreased in *mpv17^a9/a9^* but not *pappaa^p170^* mutants. As Ca^2+^ is involved in intracellular signaling, it is also possible that other cellular processes such as transcription or intracellular transport could be altered in *mpv17^a9/a9^* mutants, leading to changes in expression, assembly, or stability of mechanotransduction channel components. Given that neomycin uptake is likely reduced in *mpv17^a9/a9^* mutant hair cells (Hailey et al., 2017), our observations support that mitochondrial dysfunction is the main source of increased vulnerability to neomycin. In *pappaa^p170^* mutants, this susceptibility was rescued by treatment with the ROS scavenger mitoTEMPO, suggesting that attenuating oxidative stress could rescue *mpv17^a9/a9^* susceptibility to neomycin. Additionally, a study defining neomycin-induced hair-cell death showed that mitochondrial Ca^2+^ uptake in zebrafish neuromast hair cells is a precursor to cell death, so it is possible that the elevated mitochondrial Ca^2+^ observed in *mpv17^a9/a9^* hair cells may contribute to the heightened sensitivity (Esterberg et al., 2014). This study also demonstrated that ΔΨ_m_ plays a role in neomycin ototoxicity such that partially depolarizing the mitochondria with sub-lethal levels of FCCP offered a protective effect. Cumulatively, our observations in *mpv17^a9/a9^* hair cells provide additional support to the idea that mitochondrial dysfunction underlies enhanced susceptibility to neomycin-induced hair-cell death, and that drugs targeting ROS or that uncouple mitochondrial phosphorylation may provide therapeutic protection.

In larvae exposed to strong water current stimulus, *mpv17^a9/a9^* neuromasts displayed mechanical disruption of morphology more frequently than WT siblings exposed to the same stimulus (Figure 5). We also observed, in disrupted neuromasts, a significant decrease in hair-cell number (Figure 6). In our previous study, we reported intact mechanotransduction was not required for stimulus-induced displacement of neuromasts, indicating this observed phenotype is a result of mechanical injury (Holmgren et al., 2021). While it is unclear why *mpv17^a9/a9^* neuromasts are more susceptible to mechanically induced displacement, it is possible that mitochondrial dysfunction in hair cells of disrupted *mpv17^a9/a9^* neuromasts sensitizes them to mechanically induced damage. It would be interesting to know whether pharmacological manipulation of ΔΨ_m_ could affect a neuromast’s vulnerability to mechanical injury and hair-cell loss. As *mpv17^a9/a9^* hair cells also showed elevated ROS (Figure 1), oxidative stress could also play a role in morphological disruption and hair-cell loss. Further examination of ROS levels in mechanically overstimulated hair cells will be important to fully understand these mechanisms.

### Mechanical Overstimulation and Hair-Cell Synapse Loss

It has been established that moderate noise exposure results in a loss of hair-cell synapses in the cochlea, but the mechanisms underlying noise-induced synapse loss are not completely understood (Kujawa and Liberman, 2009). Recent studies have defined the roles of mitochondrial Ca^2+^ in synaptic maintenance and noise-induced synapse loss (Wang et al., 2018; Wong et al., 2019). One mechanism by which Ca^2+^ is taken up by mitochondria is *via* the mitochondrial Ca^2+^ uniporter (MCU; Wong et al., 2019). Both inhibition of MCU by Ru360 and genetic deletion of MCU have been shown to prevent hair-cell synapse loss in noise-exposed mice (Wang et al., 2018). These observations support that mitochondrial Ca^2+^ uptake plays a critical role in noise-induced synapse loss. We have shown here that *mpv17^a9/a9^* hair cells have elevated mitochondrial Ca^2+^ levels, as measured by the genetically encoded mitochondrial Ca^2+^ indicator MitoGCaMP3 (Figure 1; Esterberg et al., 2014). It would therefore be unsurprising if elevated mitochondrial Ca^2+^ in *mpv17^a9/a9^* hair cells contributed to more severe hair-cell synapse loss following mechanical overstimulation. However, here we did not see a greater degree of synapse loss in overstimulated *mpv17^a9/a9^* hair cells. Instead, we observed similar degrees of synapse loss in both WT and *mpv17^a9/a9^* stimulus exposed neuromasts (Figure 8), suggesting an alternate mechanism is at play.

It has recently been shown that *Vglut3^−/−^* null mutant mice do not lose hair-cell synapses following noise exposure, supporting a role for synaptic transmission in noise-induced synapse loss (Kim et al., 2019). Additionally, in our previous study of mechanical injury in the zebrafish lateral line, we observed significantly more severe mechanically-induced hair-cell synapse loss in fish when glutamate clearance from the synapse was pharmacologically blocked, suggesting synapse loss can be exacerbated by excess glutamate in the synaptic cleft (Holmgren et al., 2021). Our results here show that *mpv17^a9/a9^* hair cells have reduced FM1-43 uptake indicating reduced hair-cell transduction (Figure 3), which suggests synaptic transmission may also be impaired in *mpv17^a9/a9^* hair cells. It is possible that there is a balancing act in *mpv17^a9/a9^* hair cells, with elevated mitochondrial Ca^2+^ potentially tipping the scales toward more severe synapse loss, yet counter balanced by reduced hair-cell activity providing protection from glutamate excitotoxicity. Further studies will be necessary to determine the relative contribution of mitochondrial activity to mechanically induced hair-cell synapse loss.

## Conclusion

We have shown here that mitochondrial homeostasis is disrupted in *mpv17^a9/a9^* hair cells of the zebrafish lateral line. Consequently, *mpv17^a9/a9^* neuromast hair cells are more vulnerable to neomycin-induced hair-cell death but are not more susceptible to synapse loss from overstimulation. This model will be useful for future studies examining not only the relative contributions of mitochondrial function to hair-cell damage but also the roles of mitochondrial homeostasis in subsequent repair following damage.

## Data Availability Statement

The raw data supporting the conclusions of this article will be made available by the authors, without undue reservation.

## Ethics Statement

The animal study was reviewed and approved by Washington University School of Medicine Institutional Animal Care and Use Committee and in accordance with NIH guidelines for use of zebrafish.

## Author Contributions

MH and LS designed the study, performed the experiments and analysis, and wrote the manuscript. All authors contributed to the article and approved the submitted version.

## Conflict of Interest

The authors declare that the research was conducted in the absence of any commercial or financial relationships that could be construed as a potential conflict of interest.

## Publisher’s Note

All claims expressed in this article are solely those of the authors and do not necessarily represent those of their affiliated organizations, or those of the publisher, the editors and the reviewers. Any product that may be evaluated in this article, or claim that may be made by its manufacturer, is not guaranteed or endorsed by the publisher.
