# Supplementary figures and images for: Influence of Mpv17 on Hair-Cell Mitochondrial Homeostasis, Synapse Integrity, and Vulnerability to Damage in the Zebrafish Lateral Line
Source: Front Cell Neurosci. 2021 Aug 3;15:693375. doi: 10.3389/fncel.2021.693375 (PMC8369198; doi:10.3389/fncel.2021.693375)

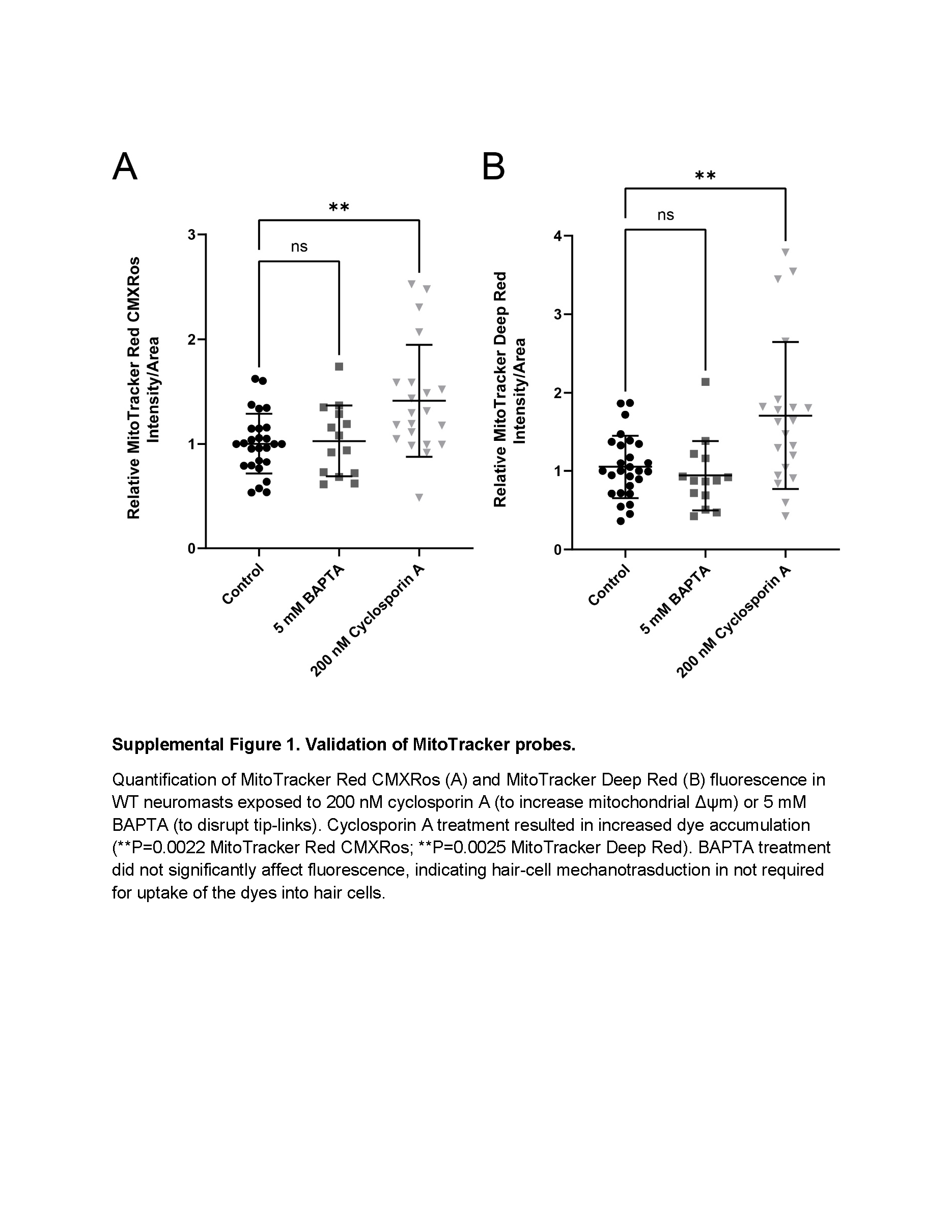

Supplement: Supplementary file 1 [file Image_1.TIF]
